# Alcohol Acyltransferase Is Involved in the Biosynthesis of C6 Esters in Apricot (*Prunus armeniaca* L.) Fruit

**DOI:** 10.3389/fpls.2021.763139

**Published:** 2021-11-11

**Authors:** Wanhai Zhou, Wenbin Kong, Can Yang, Ruizhang Feng, Wanpeng Xi

**Affiliations:** ^1^Key Lab of Aromatic Plant Resources Exploitation and Utilization in Sichuan Higher Education, Yibin University, Yibin, China; ^2^China Chongqing Agricultural Technology Extension Station, Chongqing, China; ^3^College of Horticulture and Landscape Architecture, Southwest University, Chongqing, China

**Keywords:** *Prunus armeniaca* L., alcohol acyltransferase, fruit aroma, ester, apricot

## Abstract

Short-chain esters derived from fatty acid contribute to the characteristic flavor of apricot fruit, and the biosynthesis of these compounds in fruit is catalyzed by alcohol acyltransferase (AAT). In this work, we investigated the AAT gene family via genome-wide scanning, and three AAT loci were identified in different linkage groups (LGs), with PaAAT1 (PARG22907m01) in LG7, PaAAT2 (PARG15279m01) in LG4, and PaAAT3 (PARG22697m01) in LG6. Phylogenetic analysis showed that PaAAT1 belongs to clade 3, while PaAAT2 and PaAAT3 belong to clade 1 and clade 2, respectively. In contrast, the three AAT genes present different expression patterns. Only PaAAT1 exhibited distinct patterns of fruit-specific expression, and the expression of PaAAT1 sharply increased during fruit ripening, which is consistent with the abundance of C4–C6 esters such as (*E*)-2-hexenyl acetate and (*Z*)-3-hexenyl acetate. The transient overexpression of PaAAT1 in Katy (KT) apricot fruit resulted in a remarkable decrease in hexenol, (*E*)-2-hexenol, and (*Z*)-3-hexenol levels while significantly increasing the corresponding acetate production (*p* < 0.01). A substrate assay revealed that the PaAAT1 protein enzyme can produce hexenyl acetate, (*E*)-2-hexenyl acetate, and (*Z*)-3-hexenyl acetate when C6 alcohols are used as substrates for the reaction. Taken together, these results indicate that PaAAT1 plays a crucial role in the production of C6 esters in apricot fruit during ripening.

## Introduction

Apricot (*Prunus armeniaca* L.) is a widely cultivated temperate fruit tree species with fruit containing many phytochemicals that are nutritionally valuable components of the human diet (Aubert and Chanforan, 2007). The fruits are appreciated by consumers for their unique flavor, to which aroma volatiles make an important contribution, together with sugars and organic acids (Balbontín et al., 2010; Chen J. et al., 2020). More than 300 volatiles have been identified from apricot fruit, including aldehydes, alcohols, ketones, lactones, terpenes, and esters (Cumplido-Laso et al., 2012). Among them, C6 aldehydes and alcohols offer a green-note aroma, while esters and lactones are responsible for fruity aromas. Esters such as (*E*)-2-hexenyl acetate and (*Z*)-3-hexenyl acetate are considered to be key odorants influencing the flavor quality of apricot fruit (D’Auria, 2006; Fu et al., 2017). Changes in aroma-related volatiles were previously reported during apricot fruit development and postharvest ripening (Gonzalez et al., 2009; Xi et al., 2016). Aldehydes tend to decline in the fruit while esters increase under post-harvest treatments (Cumplido-Laso et al., 2012; Galaz et al., 2013).

Volatile esters can be divided into two groups: straight-chain esters and branched-chain esters. In general, straight-chain esters are mainly synthesized via the fatty acid lipoxygenase (LOX), α-oxidation, and β-oxidation pathways (Schwab et al., 2008), while branched-chain esters are derived from the amino acid pathway. In the LOX pathway, unsaturated fatty acids, such as linoleic (18:2) and linolenic acid (18:3), are regio- and enantio-selectively deoxygenated by LOX and produce hydroperoxide isomers, which are subsequently cleaved by hydroperoxide lyase (HPL) to form hexanal and hexenal, respectively. The aldehydes can then be reduced to the corresponding C6 alcohols by alcohol dehydrogenase (ADH). Alcohol acyltransferase (AAT) catalyzes the final linkage of an acyl moiety and an alcohol to form esters and, thus, is directly responsible for the production of esters (Goff and Klee, 2006). The β-oxidation of saturated fatty acids is the primary biosynthetic process providing short-chain alcohols and acyl coenzyme A (CoA) for ester formation (Schwab et al., 2008). During the process, acyl CoAs can be reduced by acyl CoA reductase to aldehyde, which is continually reduced by ADH to alcohol. Finally, the alcohols can be used to produce esters by alcohol acyltransferase (AAT) (Bartley et al., 1985; Kruse et al., 2020; Liu et al., 2020). As for the amino acid pathway, amino acids such as L-isoleucine, L-leucine, L-valine, and L-methionine are the precursors of acyl-CoAs, involved in alcohol esterification reactions catalyzed by AATs to produce branched-chain esters (D’auria et al., 2007; Gonda et al., 2010; El Hadi et al., 2013). Therefore, alcohol acyltransferase enzymes catalyze the last decisive step in volatile ester formation in fruit (Schwab et al., 2008).

AATs from plant species belong to the BAHD superfamily of acyltransferases (ATs), which are important tailoring enzymes that contribute to the biosynthesis of several terpenoids, esters, alkaloids, and flavonoids (Kruse et al., 2020). BAHD-AT was named after the first four biochemically characterized ATs, namely benzylalcohol *O*-acetyltransferase (BEAT), anthocyanin O-hydroxycinnamoyltransferase (AHCTs), anthranilate N-hydroxycinnamoyl/benzoyltransferase (HCBT), and deacetylvindoline 4-*O*-acetyltransferase (DAT) (Bontpart et al., 2015). The proteins contain one 14–17 β-strand and one 13–17 α-helix domain connected to a crossover loop, and a solvent channel runs through the protein molecule. During the reaction, the donor binds to the front side, while the acceptor binds to the back side (Wang et al., 2021). Both of the most conserved motifs HXXXD and DFGWG are necessary for the catalytic function. HXXXD is exposed to the solvent channel and directly participates in the reaction. The catalytic His residue is accessible from both sides of the channel, while the Asp residue points away from the active site (Ma et al., 2005; Molina and Kosma, 2015). During the catalytic reaction process, the acyl acceptor and donor firstly approach the protein through the solvent channel and bind initially to the active site. Then, the basic His residue (H162) deprotonates the hydroxyl or amino group of the acceptor to form an oxyanion. Next, the oxyanion conducts a nucleophilic attack on the carbonyl carbon of the acyl donor to form a tetrahedral intermediate. Finally, the intermediate deprotonates to release a CoA-SH molecule and to form a new ester or amide (Walker et al., 2013). As for the functional protein, His-162 plays a key role in triggering the reaction, while Thr-384 and Trp-386 are vital for stabilizing the tetrahedral transition state through hydrogen bonds.

The products of AATs are mostly volatile esters, which are the main source of fruit flavors (Wang et al., 2021). During ripening of Korla fragrant pears, the production of hexyl acetate was induced and positively corelated with AAT enzyme activity and expression level of the AAT gene (Chen J. et al., 2020). A relatively high level of enzyme activity in apple fruit at harvest was observed to be accompanied by high levels of ester accumulation (Defilippi et al., 2005a). In peach, positive correlations between ester contents and AAT activity were also observed during low temperature storage plus shelf-life at 20°C (Xi et al., 2012). With increasing genome data of fruit trees, several AATs have been identified, and many AATs related to ester metabolism have been functionally characterized, such as in apple (Gonzalez et al., 2009), strawberry (Gonzalez-Agueero et al., 2009), melon (Guenther et al., 2011), papaya (Kader, 2008; Balbontín et al., 2010), and kiwifruit (Li et al., 2006). Although the role of the AAT protein in regulation of volatile ester content has been most extensively investigated in these fruits, no information is available on the function and regulation of enzymes in apricot. In our previous study, we observed that the AAT enzyme activities of apricot fruit are related to ester formation (Xi et al., 2016). However, the specific gene family member responsible for ester formation is still not identified in apricot, and the specific mechanism is poorly understood.

In the present study, we performed a genome-wide analysis of AAT family genes to identify to the specific gene impacting volatile esters in apricot fruit. PaAAT1 was ascertained as a new candidate gene through bioinformation and expression-pattern analysis. The function of cloning full-length cDNAs was verified by transient overexpression, and recombinant expression in *Escherichia coli* was used to assay the abilities of these proteins for different alcohol substrates. Ultimately, we demonstrated that PaAAT1 can function in the biosynthesis of C6 volatile esters and contribute to the aroma of apricot.

## Results

### Gene Structures and Conserved Motifs in Apricot

**FIGURE 1:**
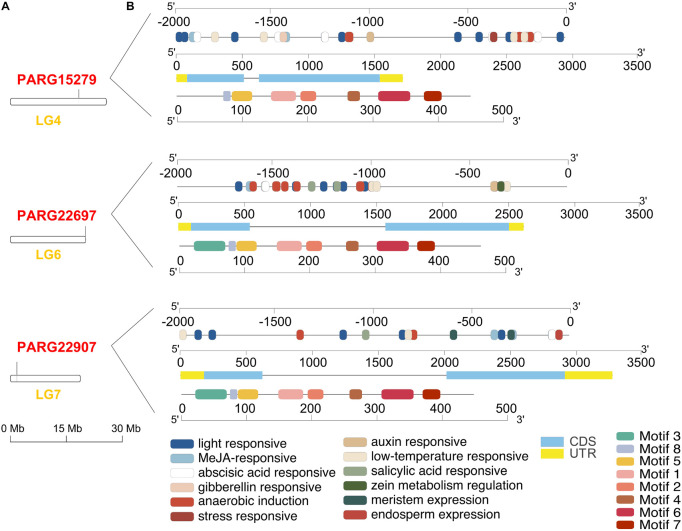
Gene structure of alcohol acyltransferase in apricot. **(A)** Distribution of three AAT genes on the different linkage group (LG) of the apricot genome. **(B)** The upper section shows the *cis*-elements distributed in the promoters of apricot AAT genes, and the rectangles of different colors represent *cis*-elements with different functions. The middle section shows the untranslated 5′- and 3′-regions (UTR), pink boxes indicate coding sequence length (CDS), and black lines denote introns. The lower section provides the motif structure of the AAT genes.

Three members of the AAT gene family were obtained from the apricot genome. Three AAT loci were identified in different linkage groups (LGs). PaAAT1 (PARG22907m01) was located in LG7, PaAAT2 (PARG15279m01) was in LG4, and PaAAT3 (PARG22697m01) was in LG6 (Figure 1A). *Cis*-acting element analysis identified a total of 12 *cis*-acting elements from three promoters (Figure 1B). Most of the *cis*-elements found were involved in the light response (TCT-motif, ACE, AE-box, G-box, Box 4, GT1-motif, ACTC-motif, GATA-motif, MIRE, and TCCC-motif). Some *cis*-elements were identified to be involved in anaerobic induction (ARE), meristem expression (CAT-box), and endosperm-specific negative expression (AACA-motif). Others were found to participate in responses to temperature (LTR), stress (TC-rich), and phytohormones, including auxin (TGA-element, AuxRR-core), methyl jasmonate (CGTCA-motif, TGACG-motif), salicylic acid (TCA-element), abscisic acid (ABRE), gibberellin (TATC-box and P-box), and auxin (AuxRR-core and TGA-element). The DNAs of PaAAT1-3 contained different numbers of nucleotides, with two exons and one intron (Figure 1B). PaAAT1-3 encodes 447, 449, and 461 amino acids, with 49.905, 50.334, and 50.936 Da, respectively. Motif analysis showed that motif 2, motif 4, motif 5, motif 6, motif 7, and motif 8 were highly conserved among all proteins and that their relative positions were nearly fixed; motif 2 was found only in PaAAT1 (Figure 1B).

### Phylogenetic Relationships and Conserved Domain Analysis

**FIGURE 2:**
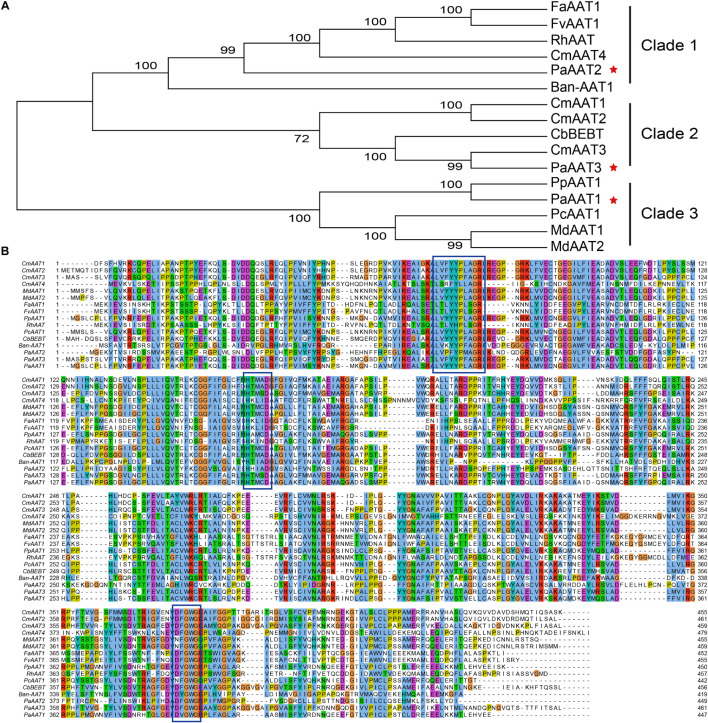
Phylogenetic analysis of the PaAAT protein and other representative alcohol acyltransferases. **(A)** Phylogenetic tree of AAT proteins. The tree is drawn to scale, with branch lengths measured by the number of substitutions per site. The percentage bootstrap values (1,000 replicates) for groupings are given for each branch. **(B)** Sequence comparison of AAT proteins. Supplementary Table 1 presents the GenBank accession numbers of each AAT sequence used. The five-pointed stars in **(A)** are the apricot AATs. The conserved motifs are framed in **(B)**.

In order to analyze the potential function, a phylogenetic tree comprising 16 AAT sequences from seven species (Supplementary Table 1) was generated showing three main subfamilies (clade 1, 2, 3) based on sequence conservation (Figure 2A), establishing a correlation between phylogenesis and functional properties of the encoded proteins. The PaAAT2 protein is clustered in clade 1 with strawberry (FaAAT1 and FaAAT1), banana (BanAAT1), and closely to melon (CmAAT4). The PaAAT3 protein is grouped in the same subfamily with melon (CmAAT1, CmAAT2) and Clarkia breweri (CbBEBT), belonging to clade 2. The PaAAT1 protein is clustered in clade 3, similar to other AATs related to the synthesis of esters in peach (PpAAT1, PcAAT1), apple (MdAAT1, MdAAT2), and closely to PpAAT1. Multiple alignment of the three putative PaAATs and other characterized AAT genes from fruit or flower highlighted the conserved motif of PaAAT1-3 proteins containing an HXXXD motif in the middle of the protein and also another conserved motif DFGWD located at the carboxylic end of each protein.

### Changes in Basic Quality Parameters and Volatile Esters During Fruit Ripening

**FIGURE 3:**
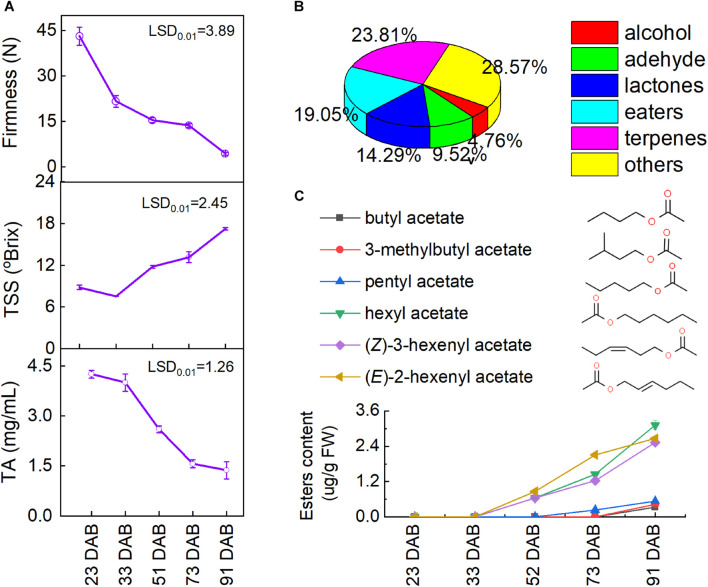
Changes in volatile esters during fruit ripening: **(A)** fruit firmness, fruit weight, total soluble solids (TSS), and titratable acid (TA) contents; **(B)** ratio of all volatiles and **(C)** esters. LSD, least significant difference (*p* < 0.01).

To evaluate the status of fruit ripening, the fruit firmness, total soluble solids (TSS), and titratable acidity (TA) were determined during fruit development and ripening. As shown in Figure 3A, the apricot fruit softened gradually from 42.2 to 4.3 N during ripening. Similarly, the TA content of tested fruit decreased significantly throughout the whole process (*p* < 0.01). Conversely, the TSS content of tested fruit increased to 17.1 ^*o*^Brix during the ripening process. Based on the changes in firmness, TSS, and TA, the sampled fruit completely covered the whole development and ripening process. During the whole development, a total of 89 volatiles were detected in the tested fruit (Figure 3B and Supplementary Figure 1), and six esters were identified from the apricots: butyl acetate, 3-methylbutyl acetate, pentyl acetate, hexyl acetate, (*Z*)-3-hexenyl acetate, and (*E*)-2-hexenyl acetate (Figure 3C). The content of these six volatile esters in tested apricots increased remarkably during ripening (*p* < 0.01), especially hexyl acetate, (*Z*)-3-hexenyl acetate, and (*E*)-2-hexenyl acetate.

### Changes in the Expression of PaAATs

**FIGURE 4:**
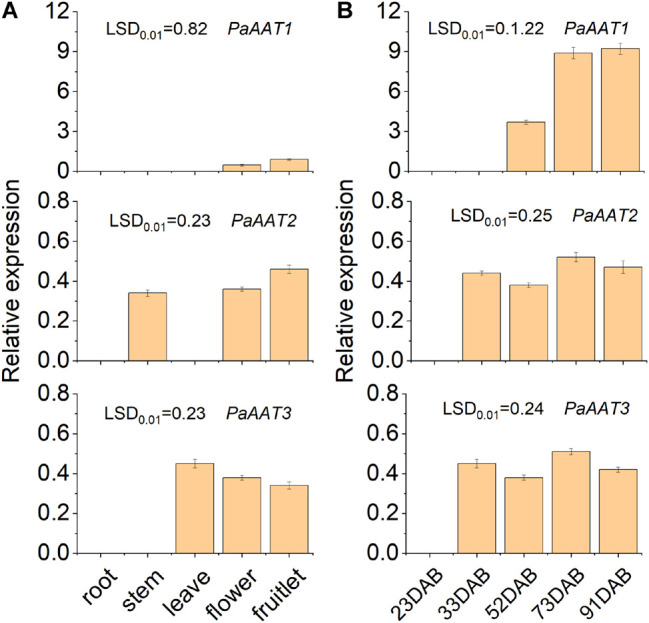
Expression of three AATs in different tissues during apricot fruit ripening. **(A)** Expression of three AATs in different tissues. **(B)** Expression of three AATs in fruit during ripening. Error bars indicate the standard error from three biological replicates. LSD, least significant difference (*p* < 0.01).

To screen out the candidate genes involved in ester formation, the transcript levels of three *PaAATs* were detected during development and ripening using quantitative real-time PCR. The difference of transcript level was observed between the three PaAATs (Figure 4). A low transcript level was observed only for *PaAAT1* in the flower and fruitlet (Figure 4A). Even *PaAAT2* was not expressed in root and leave, and a low transcript level of *PaAAT2* was detected in the stem, flower and fruitlet, while *PaAAT3* was only expressed in the leave, flower, and fruitlet (Figure 4A). As for fruit, no transcript of *PaAAT1* was detected during early development, the expression of *PaAAT1* increased remarkably during fruit ripening (*p* < 0.01) (Figure 4A), and expression of *PaAAT2* and *PaAAT3* were kept at a low level throughout the whole developmental process (Figure 4B).

### Transient Overexpression of PaAAT1 in “KT” Fruit

**FIGURE 5:**
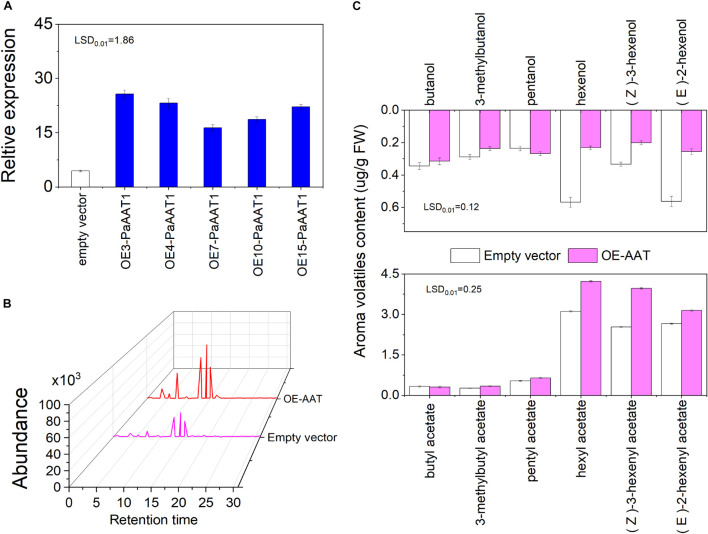
Transient expression of PaAAT1 in KT fruit; **(A)** expression of PaAAT1 infiltrated with an empty vector and PaAAT1; **(B)** chromatogram of esters in peel infiltrated with an empty vector and PaAAT1; **(C)** volatile alcohols and esters emitted from the peel injected with an empty vector and PaAAT1. LSD, least significant difference (*p* < 0.01).

Transient overexpression analyses were performed with fruit peel before the turning stage to verify the functions of PaAAT1 related to volatile ester formation in apricot fruit. The expression of *PaAAT1* in agro-infiltrated fruit was analyzed by qRT-PCR on the third day after infiltration. We found that the transcript abundance of *PaAAT1* in PaAAT1-overexpressed fruit increased by 3.68–5.77-fold compared to the control fruit (Figure 5A). Compared with the control, higher chromatographic peaks of hexyl acetate (peak 4), (*Z*)-3-hexenyl acetate (peak 5), and (*E*)-2-hexenyl acetate (peak 6) were found in PaAAT1-overexpressed fruit, but no obvious significant chromatographic peaks of butyl acetate (peak 1), 3-methylbutyl acetate (peak 2), or pentyl acetate (peak 3) were found (Figure 5B). In the PaAAT1-overexpressed fruit, the butanol, 3-methybutanol, pentanol, hexenol, (*Z*)-3-hexenol, and (*E*)-2-hexenol contents significantly decreased (*p* < 0.01) (Figure 5C); on the contrary, the hexyl acetate, (*Z*)-3-hexenyl acetate, and (*E*)-2-hexenyl acetate contents significantly increased by 30, 56, and 18%, respectively, compared to the control (*p* < 0.01), while no significant content difference of butyl acetate, 3-methylbutyl acetate, or pentyl acetate were observed between PaAAT1-overexpressed fruit and the control fruit (*p* < 0.01) (Figure 5C).

### *In vitro* Enzyme Properties and Activity of Recombinant PaAAT1

**FIGURE 6:**
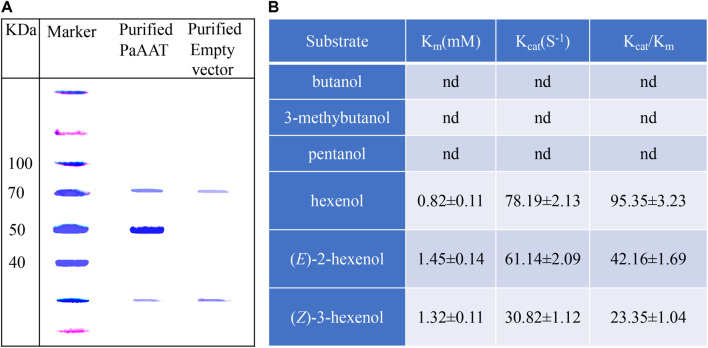
The proteins purified from *E. coli* and its enzyme activity *in vitro*. **(A)** The SDS-PAGE analysis of recombinant PaAAT proteins purified from *E. coli*; **(B)** the kinetics of properties of PaAAT1 recombinant protein. Data are presented as the means ± SE. n.d. represents not detectable.

**FIGURE 7:**
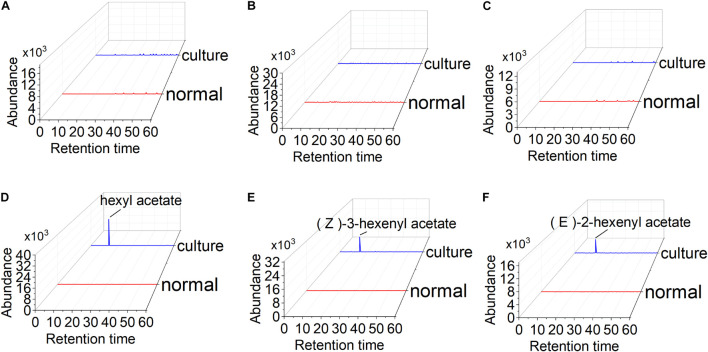
Functional analysis of the PaAAT1 enzyme *in vitro*: **(A–F)** high pressure liquid chromatography (HPLC) analysis of the esters from the incubation of butanol, 3-methybutanol, pentanol, hexenol, (*E*)-2-hexenol, and (Z)-3-hexenol with the recombinant PaAAT1 protein. Culture represents PaAAT1 protein expressed in *Escherichia coli* added to the reaction system; normal means empty vector protein was added.

To test esterized activity of PaAAT1, a recombinant PaAAT1 protein purified from *E. coli* and *in vitro* experimentation was used to feed alcohol as substrate. Headspace solid-phase micro-extraction (HS-SPME) and gas chromatography (GC) were used to determine the products. A protein of about 50 kDa was obtained (Figure 6A). The recombinant PaAAT1 protein has the highest substrate affinity (Km) with (*E*)-2-hexenol (1.45 mM), followed by (Z)-3-hexenol (1.32 mM). The highest overall activity (Kcat/Km) was observed with hexyl acetate (Figure 6B). Six alcohols including butanol, 3-methybutanol, pentanol, hexenol, (*Z*)-3-hexenol, and (*E*)-2-hexenol were used as substrates for assays to assess PaAAT protein activity for producing corresponding ester. Low levels of esters were detected when butanol, 3-methybutanol, and pentanol were used as substrates for the reaction (Figures 7A–C). However, high corresponding peaks for hexyl acetate, (*Z*)-3-hexenyl acetate, and (*E*)-2-hexenyl acetate, eluting at 12.83, 14.24, and 15.06 min, respectively, were observed when hexenol, (*Z*)-3-hexenol, and (*E*)-2-hexenol were used as the substrate (Figures 7D–F).

## Discussion

Volatile esters play an important role in determining for flavor of a number of fruits, and are positive with consumer preference (Cao et al., 2019; Qian et al., 2019). Among them, short-chain esters that are derived fatty acids are the major class of volatile organic compounds (VOCs) contributing to the overall characteristic of ripe apricots (Livak and Schmittgen, 2001; Liu et al., 2019). However, considering the range of esters found in various fruit species, a wide variety of alcohol acyltransferases with various specificities are yet to be isolated, and little is known about the genes controlling ester biosynthesis in apricot fruit. The identification of an alcohol acyltransferase responsible for ester synthesis in apricot is a key step in understanding metabolic basis for regulatory aroma quality. Recently, the release of the genome facilitated the discovery of the key genes involved in the biosynthesis of these compounds in apricot. The present study was focused on identifying the AAT genes on a genome-wide scale, as well as characterizing their functions for volatile ester formation.

Plant BAHD-ATs constitute a large family of acyl CoA-utilizing enzymes, which contribute to the diversity of natural products, including small volatile esters and modified anthocyanins, as well as constitutive defense compounds and phytoalexins (Schwab et al., 2008; Peng et al., 2020). Most of these enzymes are *O*-acetyltransferases (*O*-ATs), and a few are N-acetyltransferases. *O*-ATs are further classified according to their acceptors: alcohols, flavonoids, quinic/shikimic acids, terpenoids, alkaloids, lipids, and sucrose (Wang et al., 2021). Many plant species genomes were published, and a number of BAHD-ATs have been identified. There are estimated to be about 88 genes encoding members of the BAHD family in *Arabidopsis* (Luo et al., 2007), and Oryza sativa contains more than 120 BAHD-ATs (Kumar et al., 2021). Xu et al. (2021) identified and characterized the expression of the 46 BAHD family genes during development, ripening, and stress response in banana (*Musa acuminata* Colla). A total of 717 BAHD-AT genes were identified from seven Rosaceae species, including the genomes of *Pyrus bretschneideri*, *Fragaria vesca*, *Malus domestica*, *Prunus avium*, *Pyrus communis*, *Prunus persica*, and *Rubus occidentalis*, producing final lists of 114, 89, 140, 124, 86, 81, and 68 putative BAHD genes (Liu et al., 2020). In the present study, based on conserved motif analysis, a total of 153 BAHD-AT genes were identified from the apricot genome (Supplementary Table 2). Recently, Wang et al. (2021) summarized biochemically characterized catalytic function and mechanisms of 141 ATs in plants. Among them, only 11 ATs can utilize benzoyl-containing alcohols or short/medium-chain alcohols as acyl acceptors. These results suggested that even the BAHD-AT family contains a large number of gene members in plants, and the members of this family have some unique roles. Only several AAT paralogs exist in many plant genomes, producing diverse ester volatiles associated with fruit flavor and flower aroma.

So far, many AAT genes expressed in fruit from this family have been earlier implicated in the biosynthesis of volatile esters (Goulet et al., 2015). The expression of *SlAAT1* substantially increases during ripening of the tomato fruit, in parallel with genes encoding other volatile-related enzymes, including SlCXE1 and lipoxygenase C (Goulet et al., 2012). Correlations between expression of flavor-related AAT genes and fruit ripening have also been established in other species including melon (El-Sharkawy et al., 2005), grape (Kalua and Boss, 2009), papaya (Balbontín et al., 2010), apple (Li et al., 2006), peach (Xi et al., 2012), *Fragaria ananassa*, and *Fragaria chiloensis* (Cumplido-Laso et al., 2012). However, of the five SlAAT genes, only SlAAT1 is highly expressed in the ripe fruit. In the present study, three PaAATs were identified from the apricot genome. To demonstrate the contribution of three PaAATs to ester production in apricot fruit, their expression levels were detected by qPCR. The results revealed that only *PaAAT1* was highly expressed in fruit. Simultaneously, the expression level of *PaAAT1* increased rapidly during the fruit ripening in parallel with volatile ester production. The expression pattern of *PaAAT1* was strongly linked with the accumulation of six esters, especially with the accumulation of hexyl acetate, (*Z*)-3-hexenyl acetate, and (*E*)-2-hexenyl acetate, which suggested that *PaAAT1* might play a vital role in the synthesis of esters in apricots.

Despite the low sequence homology found among the AAT genes identified to date, AAT proteins exhibited some common characteristics. All the fruit AAT genes identified to date encode proteins ranging from 419 to 479 amino acid residues, which corresponds to an average molecular weight of 48–55 kDa (Wang et al., 2021). The present study found that PaAAT1s are globular proteins with a size of 49.905 kDa, which is in good agreement with the molecular weight of the native AAT proteins purified in banana (40 kDa), strawberry (48 kDa), grape (50 kDa), and melon (50 kDa) (Hui et al., 2010). As for the complete protein, conserved motifs are generally the basis for recognition of homologous proteins across species boundaries. Most functionally characterized BAHD acyltransferases share two conserved motifs: a conserved HXXXD(G) motif and a less conserved DFGWG motif (D’Auria, 2006; Unno et al., 2007). For VlAMAT of the Concord grape cultivar, however, besides the basic motifs, another conserved LXXYYPXAGR motif existed near the N terminus of the sequence (75–84 residues). In the present study, PaAAT1 not only contained two common HTMCD (165–169 residues) in motif 1 and DVGWG (386–340 residues) conserved motifs in motif 7 but also contained the AAT-specific conserved motif LVYYYPLAGR (74–83 residues) in motif 8 (Figures 1, 2). Even so, residue differences might significantly alter the catalytic activity of the AAT protein and dramatically affected the esterification reactions as reported for SAAT, VAAT, CmAAT1-4, and peach (D’Auria, 2006; Song et al., 2021). Based on this, we inferred that the difference of two conserved motifs HXXXD and LXXYYPXAGR for position X might be a main reason for the difference in esterification ability of PaAAT1-3.

Plants are diverse in volatile esters, and the diversity mainly depends on substrate specificities of their corresponding proteins. Similar to BAHD enzymes, most of the native and recombinant AAT proteins exhibit broad specificities of many substrates. In many fruits, different specificities of the multiple AAT proteins codified by the genes have been identified. In strawberry, FaAAT2 and FvAAT1 both influence the ester content of the fruit. FaAAT2 has been identified to be responsible for ester biosynthesis in cultivated strawberry (*Fragaria* × *ananassa*); the enzyme had activity with C1–C8 straight-chain alcohols and aromatic alcohols in the presence of acetyl-CoA (Cumplido-Laso et al., 2012), whilst FvAAT function in wild strawberry (*Fragaria vesca*) and the FaAAT2 enzyme have a preference for C6–C10 aliphatic alcohols (Beekwilder et al., 2004). In apple (*Malus pumila*), at least two AAT enzymes are thought to produce ester volatiles (Souleyre et al., 2005; Li et al., 2006), and silencing AAT1 results in a strong decrease in some individual compound levels of propyl and butyl 2-methylbutanoate as well as propyl propanoate in ripe fruit (Souleyre et al., 2014; Yauk et al., 2017). Overexpressing MdAAT2 in transgenic tobacco leaves significantly increased the concentrations of methyl benzoate and methyl tetradecanoate, suggesting that MdAAT2 may use medium-chain fatty acyl CoA and benzoyl-CoA as acyl donors together with methanol acceptors as substrates (Li et al., 2008). Melon CmAAT1, CmAAT3, and CmAAT4 effectively account for the great diversity of esters formed in the fruit (El-Sharkawy et al., 2005; Galaz et al., 2013). As an aromatic alcohol AT, PhBPBT in *Petunia hybrida* displayed a broad spectrum for 13 acceptors, including: benzyl alcohol, 3-hydroxybenzyl alcohol, and 2-phenylethanol (Wang et al., 2021). PtSABT and PtBEBT from *Populus trichocarpa* catalyzed the formation of salicyl benzoate and benzyl benzoate with 75 and 70% conversion rates, respectively (Wang et al., 2021). All these examples illustrate the specificity in aroma production that each species can acquire while maintaining an overall ability to produce an array of esters. Peach PpAAT1 can produce esters and lactones by different acetyl (Song et al., 2021). In the present study, phylogenetic analysis revealed that PpAAT1 was close to PpAAT1, and overexpression PaAAT1 led to the remarkable content increase in hexyl acetate, (*Z*)-3-hexenyl acetate, and (*E*)-2-hexenyl acetate. Therefore, PpAAT1 might be the most important enzyme for volatile C6 ester biosynthesis in apricot fruit.

Previous work suggested that volatile esters or the expression of AAT genes is influenced by plant hormone treatment and specific transcription factors. The expression of genes from wild and cultivated strawberries (Aharoni et al., 2004), banana (Beekwilder et al., 2004), melon (El-Sharkawy et al., 2005), and grape (Wang and De Luca, 2005) is strongly induced during fruit ripening. Ethylene has proved to be a major regulator of the AAT activity in apple (Defilippi et al., 2005b; Schaffer et al., 2007), melon (Flores et al., 2002), kiwifruit (Zhang et al., 2009; Günther et al., 2015), pear (Li G. et al., 2014) and peach (Ortiz et al., 2010). Abscisic acid is also reported to be involved in aromatic ester biosynthesis related to ethylene in green apples (Wang et al., 2018). Salicylic acid and methyl jasmonate enhance ester regeneration in 1-MCP-treated apple fruit after long-term cold storage (Li et al., 2006). The expression of apple AAT (MdAAT2) is induced by salicylic acid (SA) in apple fruits and the MdAAT2 promoter’s response to SA and ethylene in transgenic tobacco (Li P.-C. et al., 2014). Influence of methyl jasmonate (MeJA) on production of aroma-related esters in “Nanguo” pears was recently closely connected to ethylene biosynthesis and signal transduction (Yin et al., 2021). In the present study, an ABA, MeJA, and salicylic acid-response element (TCA) was found in the promoter of PaAATs (Figure 1B), suggesting that the three plant hormones might play a potential regulatory role in PaAAT expression and the ester biosynthesis pathway. At present, only few transcription factors have been found to regulate ester formation and AAT expression. In strawberry, overexpression of FveERF could activate the expression of the AAT gene and ester accumulation (Li et al., 2020), and volatile esters in postharvest strawberry were affected by the interaction of light and temperature (Fu et al., 2017). In the study, *cis*-elements of light response and temperature were found in the promoters of three PaAAT1s, suggesting that PaAAT1s are also involved in light and temperature regulation. These studies suggested that the ester metabolism was finely controlled by the complex network of environment factors, transcription factors, and AAT expression.

## Materials and Methods

### Identification of Alcohol Acyltransferase Gene Family Members and Chromosomal Distribution

Sequences and annotations of the apricot (*Prunus armeniaca*) genome were obtained from Genome Database for Rosaceae (GDR).^1^ We extracted the AATase domain (PF07247) from the Pfam database^2^ to determine the AAT proteins using the HMMER 3.0 software (Finn et al., 2011). The *E*-value of the HMMER search results should be less than 0.001. Whether candidates could be the AAT gene family members was contingent on whether they had the conserved AAT domain, so Pfam^3^ (Bateman et al., 2004), NCBI^4^, or SMART^5^ (Letunic et al., 2012) were required to examine the presence of the conserved AAT domain. All AAT genes were mapped to the chromosomes of apricot using TBtools (Chen C. et al., 2020).

### Gene Structure Analysis and *Cis*-Element Prediction

The exon–intron structure of the AAT genes was obtained via the online tool Gene Structure Display Server (GSDS)^6^. MEME^7^ was used to analyze the conserved motifs under the following parameters: motif site distribution with any number of repetitions and 20 as the maximum number of motifs. Other parameters used the default settings. The results were visualized using TBtools (Chen C. et al., 2020). The promoter sequences (2 kb upstream of the 5′ untranslated regions (UTR)) for all AAT genes were used for *cis*-element identification via submission to the PlantCARE database.^8^ The molecular weight (MW) of the AATs obtained was calculated using the ExPASy Compute pI/MW Program.^9^

### Phylogenetic Analysis

The full-length apricot AAT protein sequences were obtained from the Genome Database for Rosaceae (see text footnote 1), and the amino acid sequences and accession numbers of AAT in strawberry, melon, banana, peach, Clrikia breweri, apple, papaya, and *P. communis*, as described (Yauk et al., 2017), were downloaded from GenBank.^10^ The related sequence information is shown in Supplementary Table 1. The alignment of all sequences was performed with the MEGA version 10.1 software. The aligned result was used to construct a phylogenetic tree via the neighbor-joining (NJ) method with 1,000 bootstrap replicates. The results were visualized by the online tool iTOL^11^ (Chen C. et al., 2020).

### Plant Materials

The cultivar Katy apricot (KT) was grown in Liuchuan town, Jingyuan county, Bainyin, Gansu, China. “KT” apricot trees were planted in 2014 in rows in a north–south orientation, with a distance of 3-4 m between rows. Normal management and pest control were uniformly carried out according to local practices. Fifteen individual trees were marked; five trees were considered as one replicate, with a total of three biological replicates. Roots, leaves, and flowers were collected at the flowering phase. Fruits were harvested 23, 33, 51, 73, and 91 days after blossoming (DAB). After being transported to the laboratory on the day of picking, 90 fruits of uniform size and free of visible defects were collected at each stage between April 20th and August 10th; 20 fruits were used as one biological replicate. For each replicate, 10 of the 20 fruits were used to measure basic physiological indexes such as the firmness, total soluble solids (TSS), and titratable acidity (TA). After measuring the basic quality values, all materials were immediately frozen in liquid nitrogen and kept at −80°C until use.

### Fruit Ripening Evaluation

A texture analyzer (TA-XT2i Plus, Stable Micro System, United Kingdom) fitted with a 7.9 mm diameter head was used to measure the fruit firmness according to the method of described by Xi et al. (2016). The rate of penetration was 1 mm s^–1^ with a final penetration depth of 10 mm. Two measurements were made on opposite sides at the equator of each fruit after the removal of a 1 mm-thick slice of skin. Ten fruits were considered one replicate, and three replicates were used for each sampling point. To determine the total soluble solids (TSS) values, three drops of juice from each slice were applied to an Atago PR-101α digital hand-held refractometer (ATAGO, Tokyo, Japan). The TSS value was read as the degree (°) Brix of the juice. Titratable acidity (TA) values were determined after the juice sample was diluted 100 times with pure water. The solution was transferred into a 250 mL beaker, which was placed over a magnetic stirrer to provide a continuous stirring of the sample solution. A pH meter probe was then immersed into the solution, and 0.1 N NaOH was added until the pH of the sample exceeded 8.1. TA was expressed in mg citric acid mL^–1^ pulp juice. All measurements were performed in triplicate.

### Volatile Ester Analysis

Volatile esters were determined according to our previous method (Xi et al., 2012). Five grams of frozen flesh powder were homogenized with saturated NaCl solution and then incubated at 40°C for 30 min. Before the vials were sealed, 30 μL of 2-octanol (8.69 mg mL^–1^) was added as an internal standard and stirred for 10 s with a vortex. For solid-phase microextraction (SPME) analysis, samples were equilibrated at 40°C for 30 min, and then volatile esters were extracted using a SPME needle with a 1 cm-long fiber coated with 65 μm divinylbenzene/carboxen/polydimethylsiloxane (DVB/CAR/PDMS) fibers (Supelco Co., Bellefonte PA, United States). The volatiles were subsequently desorbed over 5 min into the splitless injection port of the GC (Agilent 6890N equipped with a DB-WAX column, 0.32 mm, 30 m, 0.25 μm, J&W Scientific, Folsom CA, United States). The separation conditions were as follows: injector 230°C, initial oven temperature 34°C held for 2 min, increased by 0.033°C s^–1^ to 60°C, then increased by 0.083°C s^–1^ to 220°C and held for 2 min. Nitrogen was used as a carrier gas at 16.7 L s^–1^. Volatiles were identified by comparison of retention times with those of authentic standards from Sigma-Aldrich, and their contents were quantified based on the standard curves of authentic compounds. Nitrogen was used as a carrier gas at a flow rate of 1 mL min^–1^. Three biological replicates were used for each sample.

### Real-Time Quantitative PCR Analysis

Total RNA was isolated and extracted from 1 g of fruit for each sample using a Tiangen reagent kit (Tiangen, Beijing, China). Contaminating genomic DNA was removed by RNase-free DNase I (Fermentas) treatment. The concentration of isolated total RNA was determined by the absorbance at 260 nm (A260) using a NanoDropND-3300 fluorospectrometer with Quant-iTRibo GreenRNA reagent (Invitrogen) following the instructions of the manufacturer, and the integrity was evaluated by electrophoresis on 1.0% agarose gels. The cDNA was synthesized from 3.0 μg of DNA-free RNA with RevertAid Premium reverse transcriptase (Fermentas) and oligo d(T)18 as primers followed the protocol of the manufacturer. Ribosomal RNA and actin gene expression were used as the normalization reference. Specific primers were designed using Primer5 (Supplementary Table 3). Gene expression levels were detected using an iQ5 instrument (Bio-Rad Laboratories, Inc., America) with a SYBR^®^ Premix Ex Taq^TM^ II Kit (TaKaRa Biotechnology (Dalian) Co, Ltd., China). The amplification program was as follows: 95°C for 1 min, followed by 40 cycles at 95°C for 20 s, 58°C for 20 s, and 72°C for 30 s. Each qRT-PCR analysis was performed in triplicate, and the mean value was used for the qRT-PCR analysis. The relative expression of the genes was calculated according to the 2 –ΔΔCT method (Zhang et al., 2019).

### Transient Overexpression in Apricot Fruit

The overexpression pMDC32 binary vector 49 was constructed according to the method described by Liu et al. (2019). The PaAAT1 coding sequence was amplified by PCR using the primers 5′ F: ATGGGTTCATTGTGCCCTCTA 3′ (forward) and 5′ CTCCTCGACATGCTCCAATGT 3′ (reverse) and cloned using a pCR8^TM^/GW/TOPO TA cloning kit (Invitrogen). After the sequence was confirmed, the PCR product was inserted into the pMDC32 binary vector49 using Gateway LR Clonase II Enzyme mix (Invitrogen), thereby generating the overexpression vector, which was transformed into *Agrobacterium tumefaciens* GV3101. The transformed *A. tumefaciens* strain was cultured in a liquid MS medium to a final O.D. of 0.8. One mL of the suspension was then evenly injected into the “KT” fruit before the turning stage (51 days post anthesis). As a control, fruits were injected with *Agrobacterium tumefaciens* carrying an empty vector. Thirty fruits were injected with *A. tumefaciens* of PaAAT1 overexpression vector, and 10 were injected with the empty. Ester levels were measured 7–14 days after injection according to the abovementioned method. Three biological replicates were used for each sample.

### *In vitro* PaAAT1 Enzyme Assay

The *in vitro* PaAAT1 protein expression and enzyme assay was performed according to the method described by a previous study (Cumplido-Laso et al., 2012). The full-length PaAAT1 cDNA from “KT” was amplified by RT-PCR and cloned into the bacterial expression vector pEXP5-CT/Topo (Invitrogen, California, United States) and was expressed in *Escherichia coli*. For recombinant PaAAT1 protein expression, 300 μL of transformed *E. coli* cells were cultured in fresh Luria–Bertani medium (300 mL) supplemented with ampicillin (μg/mL) at 37°C until OD600 = 0.6. The expression was induced with 1 mM isopropyl-b-D-thiogalactoside. After 12 h of growth at 16°C, cells were harvested by centrifugation, and the pellet was frozen for 15 min at −80°C. After suspension, the cells were sonicated on ice. The sonicated cells were centrifuged at 4°C and 20,000 × g for 20–30 min. The soluble protein fraction (supernatant) was incubated with GST-sepharose (Novogen) for at least 30 min at 4°C with continuous agitation and then centrifuged at 4°C and 800 × g for 3 min. The protein attached to the sepharose was washed and the sepharose-bound protein was released by incubating for 5 min in 200 μL of 1 × elution buffer at room temperature and centrifuged again at 800 × g for 5 min. The eluate was quantified by Bradford’s method (Bradford, 1976) and analyzed by SDS-PAGE. The activity of recombinant semi-purified FaAAT2 protein was quantified by its ability to convert different alcohols and acyl-CoA substrates into the corresponding esters. PaAAT1 protein (2 μg) in 500 μL of total volume was used in the presence of 20 mM alcohol and 0.1 mM acyl-CoA in 50 mM TRIS-HCl buffer (pH 7.5) containing 10% (v/v) glycerol and 1 mM dithiothreitol (DTT). (*E*)-2-hexenol, (*Z*)-3-hexenol, hexenol, pentanol, 3-methybutanol, and butanol were used for the substrate test. Reactions were always initiated by adding semi-purified recombinant PaAAT1 protein and then incubated with continuous agitation at 30°C for 20 min. After incubation, 1 mM nonane was added as internal standard. The volatiles was extracted and analyzed with SPME-GC as described above. At least three biological replicates were performed for enzymatic assays.

## Conclusion

In the study, three AAT genes were identified in the apricot genome. The gene structure and gene expression analysis indicated that *PpAAT1* is putatively associated with ester formation in apricots. The transient overexpression of PaAAT1 in apricot fruit resulted in a remarkable decrease in hexenol, (*E*)-2-hexenol, and (*Z*)-3-hexenol levels while significantly increasing the corresponding acetate production (*p* < 0.01). The substrate assay revealed that the PaAAT1 enzyme can produce hexenyl acetate, (*E*)-2-hexenyl acetate, and (*Z*)-3-hexenyl when C6 alcohols are used as substrates for the reaction. These results suggest that PpAAT1 is responsible for the biosynthesis of C6 esters in apricot during fruit ripening. However, the regulation mechanism of esters needs to be further explored in the future work.

## Data Availability Statement

The original contributions presented in the study are included in the article/Supplementary Material, further inquiries can be directed to the corresponding author/s.

## Author Contributions

WX conceptualized the experiments, reviewed, and edited the manuscript. WZ and WK performed the experiments. CY analyzed the data. WZ and RF prepared the manuscript. All authors contributed to the article and approved the submitted version.

## Conflict of Interest

The authors declare that the research was conducted in the absence of any commercial or financial relationships that could be construed as a potential conflict of interest.

## Publisher’s Note

All claims expressed in this article are solely those of the authors and do not necessarily represent those of their affiliated organizations, or those of the publisher, the editors and the reviewers. Any product that may be evaluated in this article, or claim that may be made by its manufacturer, is not guaranteed or endorsed by the publisher.

## Footnotes

1
https://www.rosaceae.org/

2
http://pfam.xfam.org/

3
http://pfam.xfam.org/search#tabview=tab1

4
https://www.ncbi.nlm.nih.gov/Structure/cdd/wrpsb.cgi

5
http://smart.embl-heidelberg.de/

6
http://gsds.cbi.pku.edu.cn/

7
http://meme-suite.org/tools/meme

8
http://bioinformatics.psb.ugent.be/webtools/plantcare/html/

9
https://web.expasy.org/compute_pi/

10
https://www.ncbi.nlm.nih.gov/

11
https://itol.embl.de/index.shtml
