# Supplementary material for: Alcohol Acyltransferase Is Involved in the Biosynthesis of C6 Esters in Apricot (Prunus armeniaca L.) Fruit
Source: Front Plant Sci. 2021 Nov 11;12:763139. doi: 10.3389/fpls.2021.763139 (PMC8636060; doi:10.3389/fpls.2021.763139)

frontiers

Supplementary Material

Table S1 GenBank accession numbers of AAT sequences used in Figure 2.

Name	GenBank Accession No.	Abbreviations for species, acyltransferase (AT) names
CmAAT4	AAW51126	*Cucumis melo* alcohol acyltransferase
RhAAT1	AAW31948	*Rosa hybrid* acetyl CoA geraniol/citronellol acetyltransferase
FvAAT1	AAN07090	*Fragaria vesca* alcohol acyltransferase
FaAAT1	JN089766	*Fragaria_ananassa* alcohol acyltransferase
BanAAT	CAC09063	*Musa acuminata* alcohol acyltransferase
CmAAT2	AAL77060	*Cucumis melo* alcohol acyltransferase
CmAAT1	CAA94432	*Cucumis melo* alcohol acyltransferase
CbBEBT	AAN09796	*C. breweri* benzoyl-CoA:benzyl alcohol benzoyl transferase
CmAAT3	AAW51125	*Cucumis melo* alcohol acyltransferase
PpAAT1	DY645545	*Prunus persica* alcohol acyltransferase
PcAAT1	AAS48090	*Pyrus communis* alcohol acyltransferase
MdAAT2	AAS79797	*Malus domestica* alcohol acyltransferase
MpAAT1	AY707098	*Malus pumila* alcohol acyltransferase

Table S2 Gene features of all identified BAHD genes in apricot.

	Gene ID	Chromosome	Start (bp)	End (bp)	Sequence Length(aa)	HxxxD domain	DFGWG domain
1	PARG03267m01	LG1	24034459	24038056	3597	✓	✓
2	PARG03077m01	LG1	22965874	22968617	2743	✓	
3	PARG04033m01	LG2	5259097	5260461	1364	✓	✓
4	PARG04177m01	LG2	6626451	6627815	1364	✓	✓
5	PARG05926m01	LG2	20737439	20738779	1340	✓	✓
6	PARG05921m01	LG2	20715765	20717105	1340	✓	✓
7	PARG05929m01	LG2	20748410	20749723	1313	✓	✓
8	PARG05938m01	LG2	20791377	20793469	2092	✓	✓
9	PARG05920m01	LG2	20704578	20705873	1295	✓	✓
10	PARG05898m01	LG2	20566947	20568257	1310	✓	✓
11	PARG05940m01	LG2	20819192	20820520	1328	✓	✓
12	PARG05945m01	LG2	20864833	20866161	1328	✓	✓
13	PARG05928m04	LG2	20749946	20751274	1328	✓	✓
14	PARG05950m01	LG2	20895673	20896980	1307	✓	✓
15	PARG05928m01	LG2	20748284	20751446	3162	✓	✓
16	PARG05928m02	LG2	20748284	20751446	3162	✓	✓
17	PARG05928m03	LG2	20746431	20751446	5015	✓	✓
18	PARG05939m01	LG2	20800780	20802108	1328	✓	✓
19	PARG05897m01	LG2	20564837	20566264	1427	✓	✓
20	PARG05937m01	LG2	20789639	20791004	1365	✓	✓
21	PARG07770m01	LG2	32194611	32196089	1478	✓	✓
22	PARG07769m01	LG2	32189958	32192187	2229	✓	✓
23	PARG07254m01	LG2	29173543	29176628	3085	✓	✓
24	PARG07058m01	LG2	28002419	28009209	6790	✓	✓
25	PARG07403m01	LG2	30079712	30082709	2997	✓	
26	PARG05955m01	LG2	20933366	20934553	1187	✓	✓
27	PARG05960m01	LG2	20957504	20959388	1884	✓	✓
28	PARG07769m02	LG2	32190497	32192143	1646	✓	✓
29	PARG08108m01	LG2	34419694	34422144	2450	✓	
30	PARG05914m01	LG2	20679085	20679909	824		✓
31	PARG05952m01	LG2	20909726	20910953	1227	✓	✓
32	PARG05447m01	LG2	16997300	16998942	1642	✓	
33	PARG08705m01	LG2	38601760	38603792	2032	✓	
34	PARG04317m01	LG2	7885483	7914194	28711	✓	✓

35	PARG05915m02	LG2	20681156	20681897	741		✓
36	PARG05913m01	LG2	20678600	20679034	434		
37	PARG05949m01	LG2	20890212	20891484	1272	✓	
38	PARG05915m01	LG2	20680303	20683858	3555		
39	PARG05948m01	LG2	20883172	20890154	6982	✓	✓
40	PARG11183m01	LG3	15949821	15951161	1340	✓	✓
41	PARG11177m01	LG3	15910575	15911927	1352	✓	✓
42	PARG11212m01	LG3	16051777	16053129	1352	✓	✓
43	PARG11210m01	LG3	16042902	16044847	1945	✓	✓
44	PARG11184m01	LG3	15952735	15954144	1409	✓	✓
45	PARG11180m01	LG3	15923916	15925268	1352	✓	✓
46	PARG11216m01	LG3	16072452	16073792	1340	✓	✓
47	PARG11205m01	LG3	16023694	16025040	1346	✓	✓
48	PARG11185m01	LG3	15954630	15955961	1331	✓	✓
49	PARG11202m01	LG3	16009921	16011237	1316	✓	✓
50	PARG11175m01	LG3	15899480	15900955	1475	✓	✓
51	PARG11207m01	LG3	16032718	16034064	1346	✓	✓
52	PARG11182m01	LG3	15947393	15948799	1406	✓	✓
53	PARG11201m01	LG3	16007702	16009024	1322	✓	✓
54	PARG11209m01	LG3	16039981	16041336	1355	✓	✓
55	PARG11188m01	LG3	15967002	15968836	1834	✓	✓
56	PARG11196m01	LG3	15987896	15990348	2452	✓	✓
57	PARG11195m01	LG3	15984930	15986255	1325	✓	✓
58	PARG11059m01	LG3	15124643	15126091	1448	✓	✓
59	PARG11217m02	LG3	16099625	16115037	15412	✓	✓
60	PARG11217m03	LG3	16099467	16101247	1780	✓	✓
61	PARG11217m04	LG3	16107996	16109426	1430	✓	✓
62	PARG11186m01	LG3	15958574	15959923	1349	✓	✓
63	PARG11217m05	LG3	16113619	16115037	1418	✓	✓
64	PARG11217m01	LG3	16099625	16109426	9801	✓	✓
65	PARG12128m01	LG3	22206193	22207982	1789	✓	✓
66	PARG11189m01	LG3	15969396	15971014	1618	✓	✓
67	PARG11187m01	LG3	15960905	15962308	1403	✓	✓
68	PARG11176m01	LG3	15908373	15909833	1460	✓	✓
69	PARG11198m01	LG3	15997053	15998361	1308		✓
70	PARG11179m01	LG3	15921723	15922808	1085	✓	✓
71	PARG11211m01	LG3	16049440	16050595	1155		✓
72	PARG11178m01	LG3	15920286	15920774	488		✓
73	PARG11214m01	LG3	16068190	16068911	721		

74	PARG11206m01	LG3	16029693	16031653	1960		✓
75	PARG11215m01	LG3	16069475	16069870	395		✓
76	PARG11199m01	LG3	16002652	16005368	2716		✓
77	PARG15195m03	LG4	17634097	17642368	8271	✓	✓
78	PARG15193m01	LG4	17609435	17613059	3624	✓	✓
79	PARG15279m01	LG4	18338475	18340194	1719	✓	✓
80	PARG15204m01	LG4	17728893	17732497	3604	✓	✓
81	PARG15201m01	LG4	17690942	17693293	2351	✓	✓
82	PARG16049m01	LG4	24520948	24528262	7314	✓	✓
83	PARG16049m02	LG4	24520933	24528355	7422	✓	✓
84	PARG16048m01	LG4	24505047	24506645	1598	✓	✓
85	PARG16049m03	LG4	24526382	24528355	1973	✓	✓
86	PARG16049m04	LG4	24526679	24539249	12570	✓	✓
87	PARG16051m01	LG4	24535925	24539222	3297	✓	✓
88	PARG15199m01	LG4	17651809	17653987	2178	✓	✓
89	PARG12730m02	LG4	393920	396415	2495	✓	✓
90	PARG12730m03	LG4	394192	401298	7106	✓	✓
91	PARG15203m01	LG4	17716030	17717512	1482	✓	✓
92	PARG15564m01	LG4	20691633	20693466	1833	✓	✓
93	PARG15195m01	LG4	17633651	17635725	2074	✓	✓
94	PARG16050m01	LG4	24521856	24522896	1040	✓	
95	PARG15198m01	LG4	17647415	17648846	1431	✓	
96	PARG13248m02	LG4	3236166	3250119	13953	✓	✓
97	PARG13248m01	LG4	3244042	3250119	6077	✓	✓
98	PARG13248m10	LG4	3248509	3250340	1831	✓	✓
99	PARG13248m03	LG4	3244042	3250119	6077	✓	✓
100	PARG13248m09	LG4	3244042	3245950	1908	✓	✓
101	PARG13247m01	LG4	3227746	3229716	1970	✓	✓
102	PARG13248m06	LG4	3232641	3237577	4936	✓	✓
103	PARG13245m01	LG4	3219898	3221352	1454	✓	✓
104	PARG13246m01	LG4	3223317	3224696	1379	✓	✓
105	PARG13251m01	LG4	3252902	3254320	1418	✓	✓
106	PARG13248m08	LG4	3236116	3237772	1656	✓	
107	PARG13244m01	LG4	3212201	3219301	7100	✓	✓
108	PARG13250m01	LG4	3250547	3252011	1464	✓	✓
109	PARG13248m05	LG4	3236166	3244956	8790	✓	✓
110	PARG13248m04	LG4	3232641	3244956	12315	✓	✓
111	PARG16049m05	LG4	24520948	24521613	665	✓	✓

112	PARG13244m02	LG4	3217900	3218850	950	✓	✓
113	PARG15280m01	LG4	18344640	18345059	419		
114	PARG13248m07	LG4	3232641	3236387	3746		✓
115	PARG13249m01	LG4	3238371	3239628	1257		✓
116	PARG13244m03	LG4	3218864	3219301	437	✓	✓
117	PARG13252m01	LG4	3256711	3257193	482	✓	✓
118	PARG15195m02	LG4	17635304	17635603	299	✓	✓
119	PARG16432m01	LG5	1642969	1644333	1364	✓	✓
120	PARG16434m01	LG5	1656836	1658526	1690	✓	✓
121	PARG19055m01	LG5	22520662	22522155	1493	✓	✓
122	PARG19504m01	LG5	25054800	25057536	2736	✓	✓
123	PARG16369m01	LG5	1071623	1073188	1565	✓	✓
124	PARG21735m01	LG6	12341492	12343983	2491	✓	✓
125	PARG22697m01	LG6	20124373	20126995	2622	✓	✓
126	PARG20495m01	LG6	3965445	3966878	1433		✓
127	PARG22573m02	LG6	19191504	19193283	1779		✓
128	PARG21974m01	LG6	14607354	14607743	389		
129	PARG22639m01	LG6	19673629	19674087	458		✓
130	PARG22573m01	LG6	19191915	19192241	326	✓	✓
131	PARG21973m01	LG6	14606426	14606868	442	✓	✓
132	PARG24366m01	LG7	13177426	13178988	1562	✓	✓
133	PARG22907m01	LG7	1762797	1766077	3280	✓	✓
134	PARG23476m01	LG7	6852834	6854110	1276	✓	
135	PARG26897m02	LG8	13369961	13373804	3843	✓	✓
136	PARG26894m01	LG8	13355786	13363060	7274	✓	✓
137	PARG26900m01	LG8	13380621	13381973	1352	✓	✓
138	PARG26899m01	LG8	13378512	13379882	1370	✓	✓
139	PARG25587m01	LG8	2410445	2411779	1334	✓	✓
140	PARG26896m01	LG8	13365819	13367171	1352	✓	✓
141	PARG26096m01	LG8	6808590	6809981	1391	✓	✓
142	PARG26897m01	LG8	13369728	13371547	1819	✓	✓
143	PARG26080m01	LG8	6702012	6703654	1642	✓	✓
144	PARG27969m01	LG8	20007173	20009019	1846	✓	✓
145	PARG26898m02	LG8	13374740	13376464	1724	✓	✓
146	PARG26901m01	LG8	13384938	13389135	4197	✓	✓
147	PARG25586m01	LG8	2391093	2394118	3025		✓
148	PARG26898m01	LG8	13375123	13376760	1637		✓
149	PARG26895m01	LG8	13365394	13365774	380		✓
150	PARG26748m01	LG8	12210434	12210802	368	✓	✓

151	PARG29251m01	tig00008183	630666	631314	648	✓	
152	PARG29252m01	tig00008183	631483	631854	371	✓	✓
153	PARG29493m01	tig00008194	372507	374716	2209	✓	✓

Table S3 Primer sequences for qRT-PCR.

**Primer**	**Forward primer（5′ to 3′）**	**Reverse primer（5′ to 3′）**
ACT	GTTATTCTTCATCGTCGTCTTCG	CTTCACCATTCCAGTTCCATTGTC
PaAAT1	CCCTCAGGAAACATCTTCCA	AGTTCCACCGTTGAAACGAC
PaAAT2	CCAATAGATGGACGGAACAGA	AATTGTTGGAAACCCCGTAAC
PaAAT3	TTTGGAGAGGTGGACTTTGG	AGACGTCTGAACCCCATTTG

Figure S1 Chromatograms representative of aroma volatiles from full ripe apricot fruit.

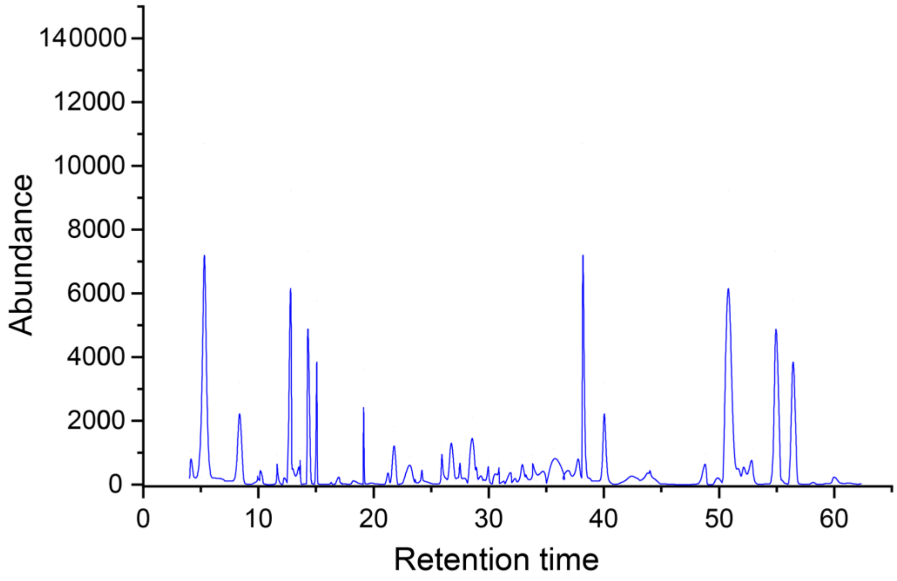

Supplement: Supplementary file 1 [file Data_Sheet_1.PDF]
